# Reversible Redox Processes in Polymer of Unmetalated Salen-Type Ligand: Combined Electrochemical in Situ Studies and Direct Comparison with Corresponding Nickel Metallopolymer

**DOI:** 10.3390/ijms23031795

**Published:** 2022-02-04

**Authors:** Julia Polozhentseva, Maria Novozhilova, Mikhail Karushev

**Affiliations:** Ioffe Physical-Technical Institute of the Russian Academy of Sciences (Ioffe Institute), 26 Polytekhnicheskaya Str., 194021 St. Petersburg, Russia; miracle_r@mail.ru (J.P.); marya20154@mail.ru (M.N.)

**Keywords:** conducting polymer, Schiff bases, salen-type ligand, *π*-stacked structures, electrochemical polymerization, UV–vis–NIR spectroelectrochemistry

## Abstract

Most non-metalized Salen-type ligands form passivation thin films on electrode surfaces upon electrochemical oxidation. In contrast, the H_2_(3-MeOSalen) forms electroactive polymer films similarly to the corresponding nickel complex. There are no details of electrochemistry, doping mechanism and charge transfer pathways in the polymers of pristine Salen-type ligands. We studied a previously uncharacterized electrochemically active polymer of a Salen-type ligand H_2_(3-MeOSalen) by a combination of cyclic voltammetry, in situ ultraviolet–visible (UV–VIS) spectroelectrochemistry, in situ electrochemical quartz crystal microbalance and Fourier Transform infrared spectroscopy (FTIR) spectroscopy. By directly comparing it with the polymer of a Salen-type nickel complex poly-Ni(3-MeOSalen) we elucidate the effect of the central metal atom on the structure and charge transport properties of the electrochemically doped polymer films. We have shown that the mechanism of charge transfer in the polymeric ligand poly-H_2_(3-MeOSalen) are markedly different from the corresponding polymeric nickel complex. Due to deviation from planarity of N_2_O_2_ sphere for the ligand H_2_(3-MeOSalen), the main pathway of electron transfer in the polymer film poly-H_2_(3-MeOSalen) is between π-stacked structures (the π-electronic systems of phenyl rings are packed face-to-face) and C-C bonded phenyl rings. The main way of electron transfer in the polymer film poly-Ni(3-MeOSalen) is along the polymer chain, while redox processes are ligand-based.

## 1. Introduction

Conducting polymers are widely used as electrode materials in sensors, electronic and optical devices, and batteries [1,2,3,4,5,6]. The introduction of transition metal centers into the conjugated backbone could significantly expand the applications and functionality of such materials. Metal-containing polymers based on transition metal complexes with Salen-type Schiff base ligands are widely studied as promising materials for many different applications [7,8,9,10,11,12,13,14,15,16,17,18,19,20,21]. The properties of these materials largely depend on substituents in the ligand environment and the metal center, special attention has been given to polymeric nickel(II) complexes with Salen-type ligands.

The mechanisms of anodic polymerization, charge transport, degradation, conductivity and other performance aspects of the polymer films have been previously described [22,23,24]. The oxidation locus in pristine nickel(II) complexes depends on the coordinating ability of the solvent, the nature of the supporting electrolyte and the presence of electron-donating or electron-withdrawing substituents in the phenyl moieties. Nickel(II) complexes with Salen-type ligands exhibit ligand-centered oxidation to phenoxyl radicals (one electron oxidation) [25] and bis-phenoxyl radical species (two electron oxidation) [26] in weakly coordinating media.

The coupling of ligand localized radicals leads to the formation of polymer films with C-C bonded molecular units [27,28,29]. Moreover, noncovalent π−π interactions between π-electronic systems of neighboring molecules are possible. In this case, π-stacks formed within the polymer film can be part of the charge transfer pathway [30,31,32]. The structure and properties of the formed films are vastly dependent on the structure of initial monomers [24].

As a result, polymer films possessing high conductivity, flexibility and stability are formed. It is recognized that the nickel metal ion does not participate in the redox processes in the polymer in weakly coordinating media, but acts as a bridge mediating charge delocalization [33]. In most cases, electrooxidation of Salen-type ligands leads to the formation of passivating films on an electrode surface. However, the ligand H_2_(3-MeSalen) could undergo polymerization forming thin polymer films [34]. There are no details of electrochemistry, doping mechanism and charge transfer pathways in the polymers of pristine Salen-type ligands in the literature, while polymers of unmetallated Salen-type ligands with polymerizable side-groups have been explored [35,36].

**Scheme 1:**
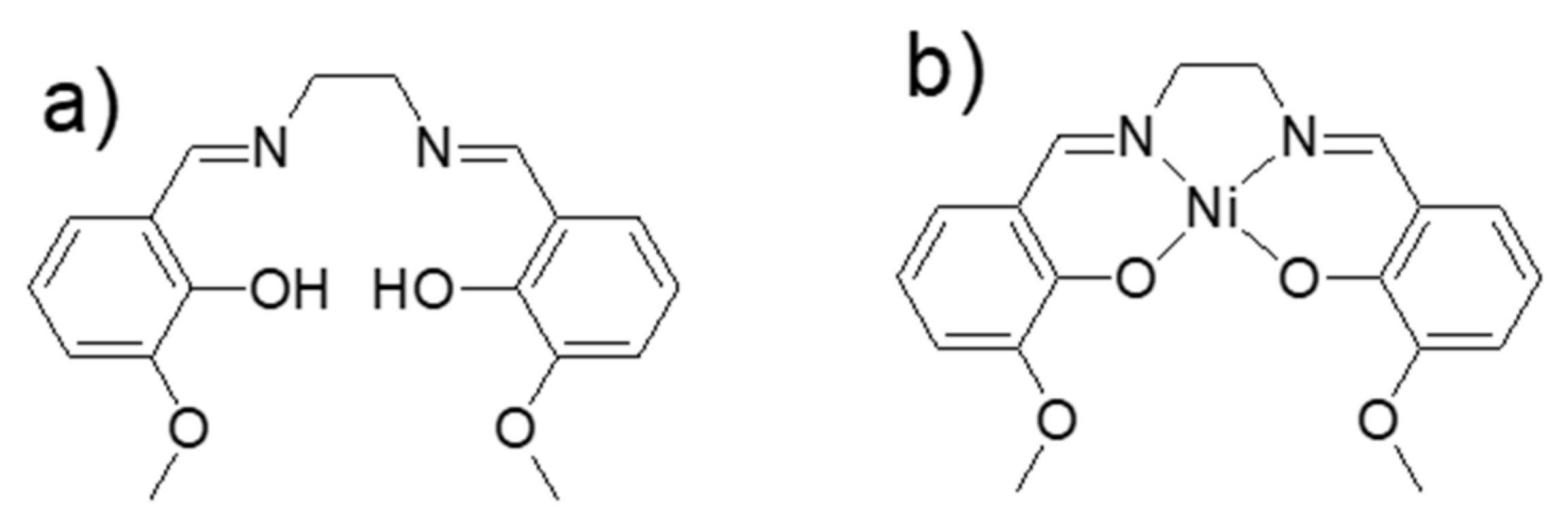
Structures of the ligand H_2_(3-MeOSalen) (**a**) and corresponding nickel(II) complex Ni(3-MeOSalen) (**b**).

Our investigations focused on the Salen-type ligand containing ethylene moiety in the imine bridge and methoxy substituents in the third position of aromatic rings, as well as its nickel(II) complex (Scheme 1).

We have discovered that the ligand H_2_(3-MeOSalen) can form electroactive polymer films similarly to the corresponding nickel complex in contrast to previous report [37]. The mechanism of Ni(3-MeOSalen) polymerization is well described, while the mechanism of ligand polymerization is obscured in the literature. The oxidation mechanism and structures of the forming films could be established by comparing electrochemical and spectroelectrochemical properties of the monomers and the corresponding polymeric films. In addition, the role of the central metal ion in the polymerization and charge transfer processes could be elucidated.

In this study, the monomeric complex and the corresponding ligand as well as the polymer films deposited on the electrode surface were investigated by cyclic voltammetry, EQCM, in situ UV–VIS spectroscopy, FTIR and scanning electron microscopy (SEM).

## 2. Results and Discussion

### 2.1. Electrochemical Polymerization of the Ligand H_2_(3-MeOSalen) and the Corresponding Nickel Complex Ni(3-MeOSalen)

**Figure 1:**
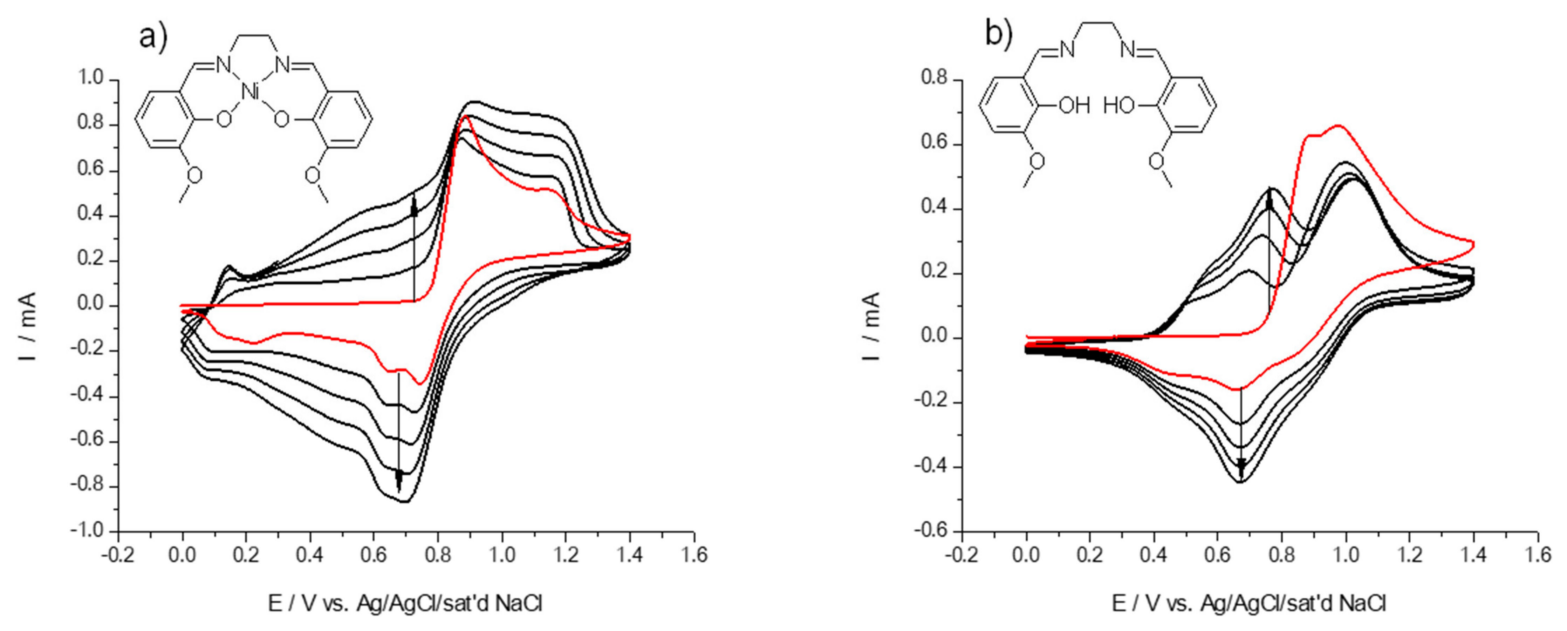
Polymerization of 0.001 mol dm^−3^ Ni(3-MeOSalen) (**a**) and 0.001 mol dm^−3^ H_2_(3-MeOSalen) (**b**) in 0.1 mol dm^−3^ Et_4_NBF_4_ acetonitrile solution, v = 0.15 V s^−1^. The first polymerization cycle is marked with red color. The arrows show the growth of the film oxidation/reduction currents from cycle to cycle.

Electrooxidation of both the ligand H_2_(3-MeOSalen) and the complex Ni(3-MeOSalen) in acetonitrile solutions leads to formation of yellow–greenish thin films on the electrode surface. Anodic polymerization occurs in monomer solution. Monomer molecules are oxidized in the near-electrode region, and the resulting polymer is deposited on the electrode in the form of a electrochemically active and electrically conductive film. In cyclic voltammograms (CV), one can observe an increase in peak currents after each subsequent cycle, which indicates the electroactive polymer film growth on the electrode surface (Figure 1a,b). This behavior is typical for nickel complexes with Salen-type ligands in weakly coordinating media [24,28,38], while most of free Salen-type ligands tend to passivate electrode surfaces during electrooxidation. In the case of H_2_(3-MeOSalen), however, the growth of an electroactive film is observed.

The first CV cycle of the nickel complex Ni(3-MeOSalen) shows two main oxidation peaks located at 0.88 and 1.13 V (Figure 1a). In previous studies, the formal potentials of 0.86 V [37] and 0.7 V [27] were reported and we suppose these correspond to the first oxidation peak observed in our study. Oxidation of Ni(ΙΙ)-Salen complexes in the absence of axial donors results in formation of the Ni(II)-phenoxyl radical electronic structure, [Ni(3-MeOSalen)]^●+^. The charge is either delocalized across the entire ligand framework or localized in one of the benzene rings [25,39,40]. The second oxidation process involves additional electron transfer yielding a bis-phenoxyl radical [Ni(3-MeOSalen)]^●●++^ [22,24]. Coupling of phenoxyl/bis-phenoxyl radicals at the *para*-positions of phenyl rings in the ligand leads to the polymer film formation on the electrode surface. Therefore, these rings become conjugated after coupling.

The H_2_(3-MeOSalen) molecule carries two hydroxyl groups. The electrochemical oxidation of phenols in aprotic solvents is accompanied by deprotonation and leads to the formation of a phenoxy radical (PhO^●^). Coupling of the phenoxy radical with another radical or the unreacted phenol molecule leads to the formation of the film consisting of phenylene oxide units. Such polymeric layers are usually electrically non-conductive, which results in surface passivation [41].

*Ortho-* and *para-* alkyl-substituted phenols undergo oxidation in organic aprotic solvents via ECE mechanism to phenoxonium cations (PhO^+^), where E is an electron transfer step, and C is a proton transfer step. One electron oxidation yields a phenoxy radical, further oxidation leads to the phenoxonium radical formation. The overall oxidation process involves the transfer of two electrons, but only one CV wave is observed because the second oxidation process occurs at less positive potentials than the first one and the intermediate deprotonation step occurs rapidly. PhO^+^ is highly reactive and undergoes self-reactions (e.g., dimerization), depending on the nature of substituents in the *ortho-* and *para-* positions. Since the oxidation of o*rtho-* and *para-* alkyl-substituted phenols is accompanied by deprotonation, it is usually irreversible and doesn’t result in the film formation [42,43].

Phenols substituted in the *ortho-* position with a nitrogen-containing group can form hydrogen bonds between hydrogen of the hydroxyl group and nitrogen atom of the substituting group. In the acetonitrile solution, the stabilization of acid form by intramolecular hydrogen bonding markedly reduces the dissociation constant [44]. Literature analysis shows that Salen-type ligands can be oxidized to phenoxyl radicals [22,45]. In the first CV cycle of the ligand H_2_(3-MeOSalen), three highly overlapping anodic peaks located at 0.86 V, 0.95 V and 1.10 V are observed (the last one is barely distinguishable).

**Scheme 2:**
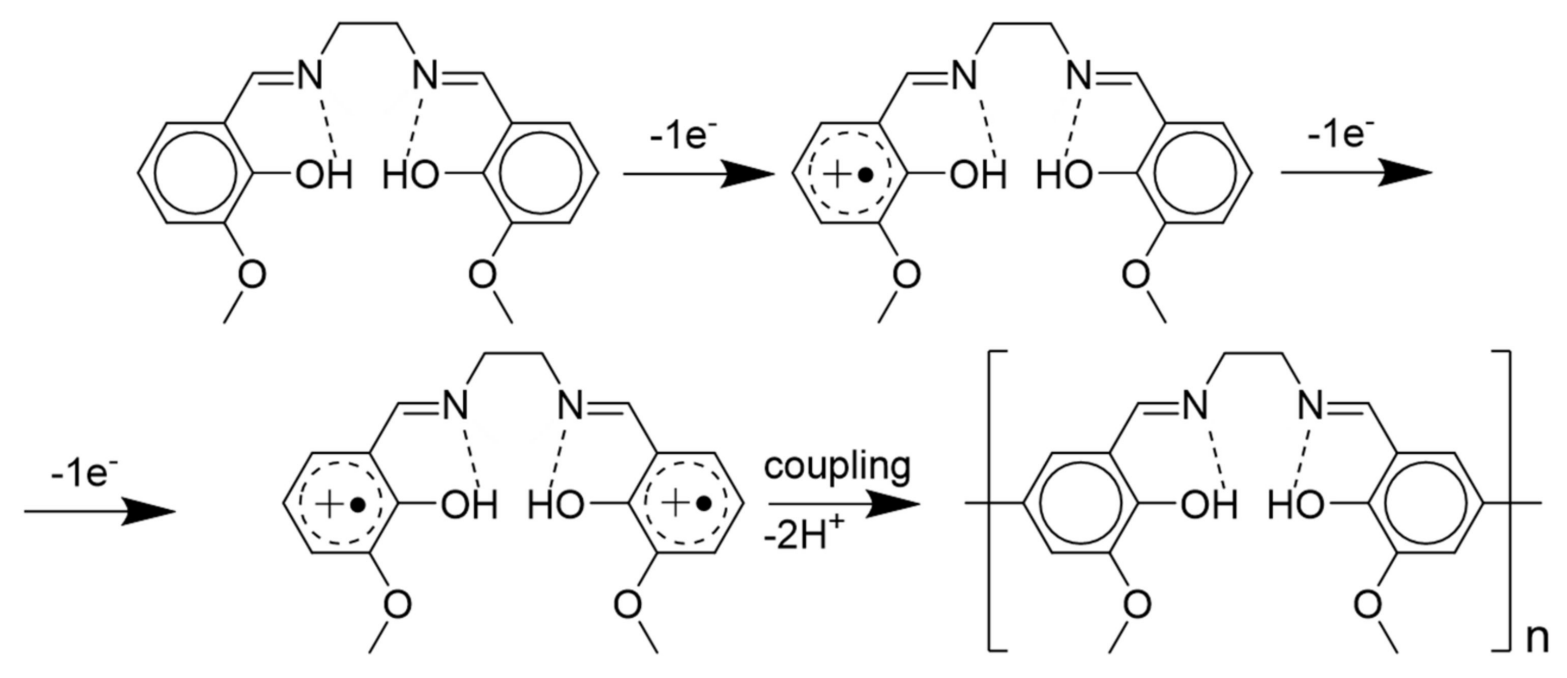
Mechanism of H_2_(3-MeOSalen) polymerization.

Similarly to the nickel complex, the ligand phenolate moiety is oxidized to the phenoxyl radical [H_2_(3-MeOSalen)]^●+^ at potentials of the first oxidation peak [46]. The bis-phenoxyl radical [H_2_(3-MeOSalen)]^●●++^ is formed during the second oxidation process. These species are stabilized not by the metal ion, but by hydrogen bonds between azomethine nitrogens and phenoxy hydrogens of the ligand molecule [47]. Coupling of phenoxyl/bis-phenoxyl radicals leads to the polymerization and film formation on the electrode surface (Scheme 2). Additionally, the formation of π-stacked structures can occur. Electronic interactions between the polymer chains have been observed in polymeric Schiff bases containing different types of transition metals [35,48]. These interactions are usually very weak and depend on the degree of charge delocalization. Similar structures can be formed in the process of H_2_(3-MeOSalen) deposition, as π-electronic systems of the phenyl rings that make up the molecules can be packed face-to-face, leading to their coupling. The structure of the polymer films will be discussed in more detail below.

### 2.2. FTIR Spectroscopy

The structures and properties of the monomeric complex of transition metals with Schiff bases are well characterized [38,49,50,51,52]. These data may be useful in the investigation of their polymer films, which are essentially conjugated systems composed of monomer units. In this work, the analysis of FTIR spectra of the polymers poly-H_2_(3-MeOSalen) and poly-Ni(3-MeOSalen) is based on their comparison with the spectra of corresponding monomers.

**Figure 2:**
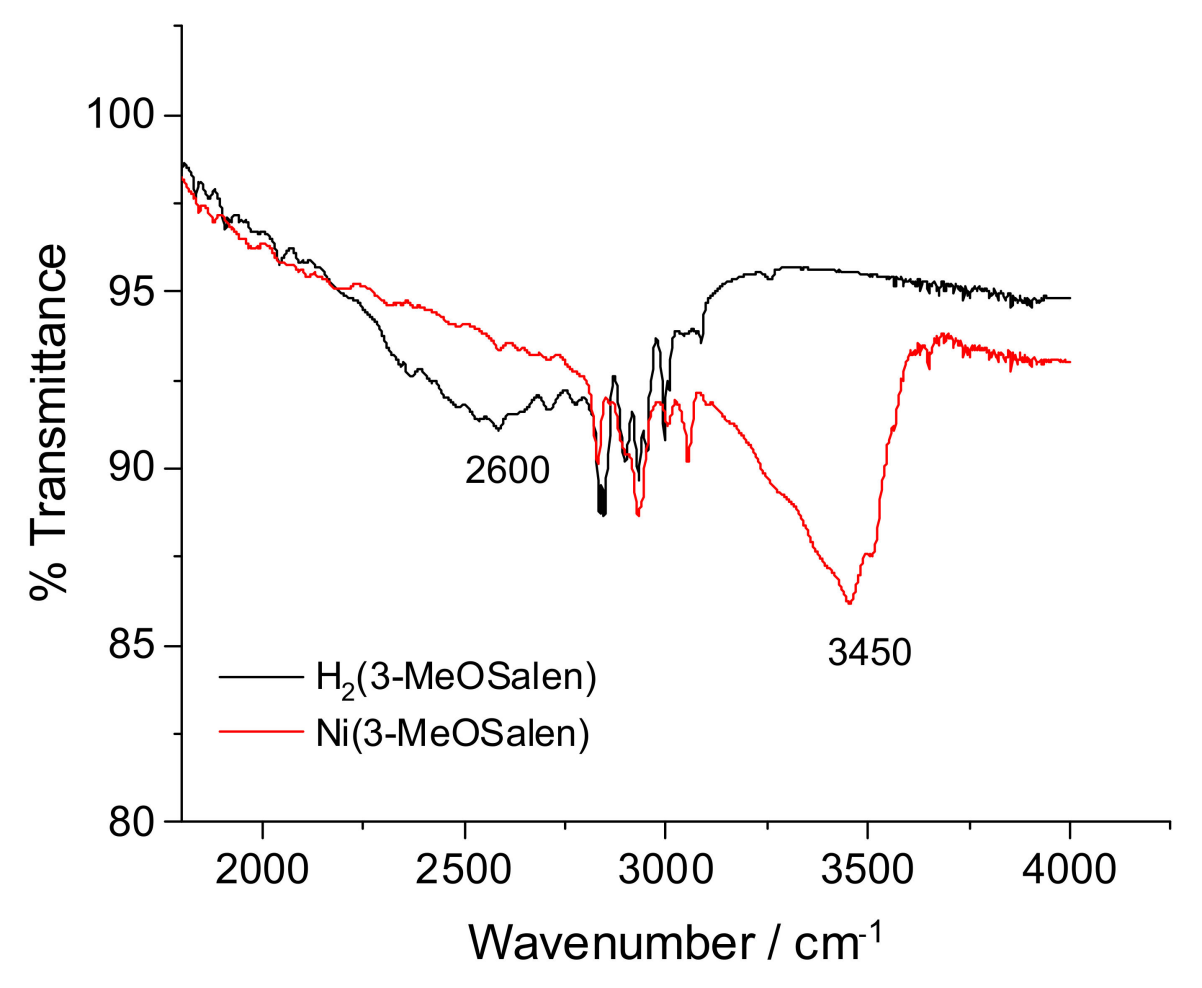
IR-spectra of the monomeric nickel complex Ni(3-MeOSalen) and the monomeric ligand H_2_(3-MeOSalen) in the frequency range of O-H stretching.

**Figure 3:**
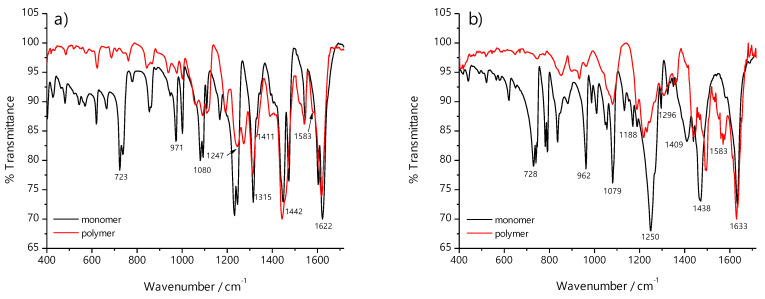
IR spectra of monomeric and polymeric nickel complexes poly-Ni(3-MeOSalen) (**a**) and monomeric and polymeric ligands poly-H_2_(3-MeOSalen) (**b**).

The most characteristic absorption bands of the monomeric ligand with methoxy substituents in the phenolate rings H_2_(3-MeOSalen) and its monomeric nickel complex Ni(3-MeOSalen) are shown in Table 1. Strong intramolecular hydrogen bonding leads to very broad and weak hydroxyl absorption (Figure 2). The O-H stretching is commonly expected in the region 3800–3500 cm^−1^, but it is dislocated to around 2600 cm^−1^ due to the internal hydrogen bridge OH…N=C. The absence of this band in the spectrum of the complex testifies to the proton substitution with the metal ion during complex formation. The azomethine (C=N) stretching is observed at 1633 cm^−1^ for the free ligand and 1622 cm^−1^ for the complex (Figure 3) [49,50,51,52]. The shift of this band by 11 cm^−1^ toward the lower frequencies in the complex points to the decrease in the bond order due to the bond formation between the azomethine nitrogen lone pair and the metal ion. The broad intensive band near 3500 cm^−1^ can be assigned to the vibrations of adsorbed water [53]. The bands corresponding to the C-O and C-N stretching frequencies are located at 1250 cm^−1^ and 1409 cm^−1^, respectively [49,50,51,52] (Figure 3). The shift of these bands to higher frequencies for the nickel complex points to the participation of nitrogen and oxygen atoms in the complexation process. Remaining IR band at 1250 cm^−1^ is related to aromatic C-C stretching located at these frequencies [52].

To elucidate the character of the bonds forming in polymer or disappearing from the monomer structure during polymerization and propose the electropolymerization mechanism, comparative analysis of the IR spectra of the polymer films and corresponding monomers has been performed.

IR spectra of the polymer complex Ni(3-MeOSalen) and its monomer are shown in Figure 3a. The main characteristic bands of the monomer spectra are found in the corresponding polymer spectra. Some of the polymer bands are slightly shifted. The shift of the bands within the range of aromatic vibrations to higher frequencies (1273(m), 1192(m), 1088(m), 976(w), 710(w) cm^−1^) corresponds to the change in the phenolate surroundings and the formation of new bonds. The presence of the band at 1583 cm^−1^ corresponding to the conjugated aromatic ring system in the film spectrum confirms the formation of a new covalent bond between aromatic rings during the polymerization process [49]. The location and intensity of the bands assigned to C=N and C-N stretching vibrations in the film (1617 and 1405 cm^−1^, respectively) remain almost unchanged. This fact excludes participation of these atomic groups in the coupling and supports the phenyl-phenyl coupling polymerization mechanism. It should be noticed that the intensity of the coordinated C-O band (1309 cm^−1^) is changed markedly, which could be related to charge delocalization and its participation in the conductive pathway.

Similar changes are observed in the ligand polymerization process for C-N, C=N and C-O bands (Figure 3b, Table 1). The behavior of the bands at ca. 1440 cm^−1^ differs comparing polymers and corresponding monomers: a shift to lower frequencies could be found for the Ni(3-MeOSalen) and to higher frequencies for the H_2_(3-MeOSalen). It is worth remarking that the band corresponding to O-H stretching (2600 cm^−1^) is present in the IR spectra of both the monomer ligand and the corresponding polymer film.

The polymerization mechanism though the formation of C-C bonds in *para*-positions of the ligand phenyl rings has been confirmed with acidic hydrolysis of different polymeric Salen-type nickel complexes [27,34]. The 4,4′-dihydroxy-3,3′-diformylbiphenyls formation was shown during acidic hydrolysis. The product yield was decreased significantly in the case of the polymeric ligand, which could mean that poly-H_2_(3-MeOSalen) film comprises fewer covalent C-C coupled phenyl moieties than the corresponding polymeric nickel complex.

### 2.3. Electrochemical Behavior of Polymer Films Poly-Ni(3-MeOSalen) and Poly-H_2_(3-MeOSalen)

**Figure 4:**
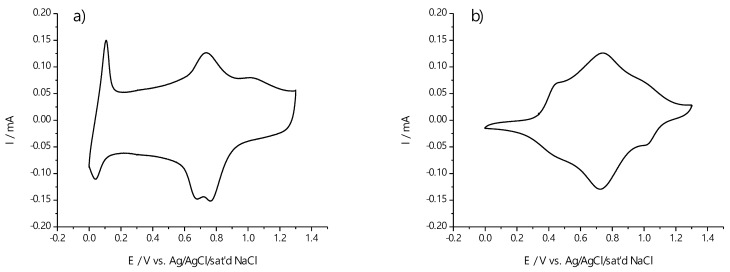
Cyclic voltammograms of the polymeric films poly-Ni(3-MeOSalen) (**a**) and poly-H_2_(3-MeOSalen) (**b**) in 0.1 mol dm^−3^ Et_4_NBF_4_/AN, 0–1.30 V, v = 0.05 V s^−1^.

The electrode coated by a polymer film was washed thoroughly and transferred into a monomer-free electrolyte solution to electrochemical studies. The cyclic voltammetry curves provide electrochemical data on redox processes occurring in the polymer. Figure 4 shows electrochemical responses of the polymer films poly-[Ni(3-MeOSalen)] and poly-[H_2_(3-MeOSalen)] in the monomer free acetonitrile solutions. All investigated polymer films show complex voltammetric responses with at least three peaks.

The cyclic voltammogram of poly-Ni(3-MeOSalen) (Figure 4a) exhibits three electrochemical processes located at 0.10 V (a sharp redox peak), 0.70 V (a broad redox peak) and ca. 1.00 V (a shoulder). Previously, *Dmitrieva* et al. [24] have investigated the electrochemical behavior and charge transfer mechanism of poly-Ni(3-MeOSalen) films in acetonitrile solution of 0.1 mol dm^−3^ Et_4_NBF_4_. It has been shown that biphenyl cation radicals [Ni(3-MeOSalen)]_n_^●+^ are formed during one electron per monomeric unit oxidation (up to 0.70 V, which corresponds to the potential of the second oxidation peak in the voltammogram). Their charge can be localized in the polymer with more electron-rich ligands or delocalized through the central nickel ion in the case of less electron-rich ligands. The methoxy-substituted Salen ligand belongs to the class of electron-rich ligands. However, the coupling between two phenolates bridged by the nickel center may still exist. The transfer of the following electron leads to the formation of biphenyl dications [Ni(3-MeOSalen)]_n_^2+^ (this process occurs mainly above 0.70 V).

The voltammogram of poly-[H_2_(3-MeOSalen)] film also reveals at least three electrochemical processes located at 0.46, 0.74 and 1.00 V (Figure 4b). Voltammetric peaks are broadened and highly overlapped, which points to several individual redox processes occurring in the same potential range. Peaks at 0.74 and 1.00 V are observed at nearly the same potentials as in the corresponding nickel complex poly-Ni(3-MeOSalen) and can be attributed to the oxidation of biphenolate fragments. The peak at 0.46 V is probably also present in the voltammogram of the polymeric nickel complex but it cannot be distinguished because of overlapping with other waves in this potential range. The peak at 0.10 V is absent in the CV of the polymeric ligand but it is present in the CV of the polymeric nickel complex. We attribute this to the effect of the nickel ion that mediates the coupling between two redox active biphenolates and induces in-chain conjugation in the polymer. The electrochemical responses of the polymeric ligand poly-H_2_(3-MeOSalen) and the corresponding polymeric complex poly-Ni(3-MeOSalen) are similar at more positive potentials. But the onset of oxidation is shifted to more negative potentials in the case of poly-Ni(3-MeOSalen), which reveals the influence of the metal-ion bridge in the coupling that allows for communication between two aromatic rings [40].

**Figure 5:**
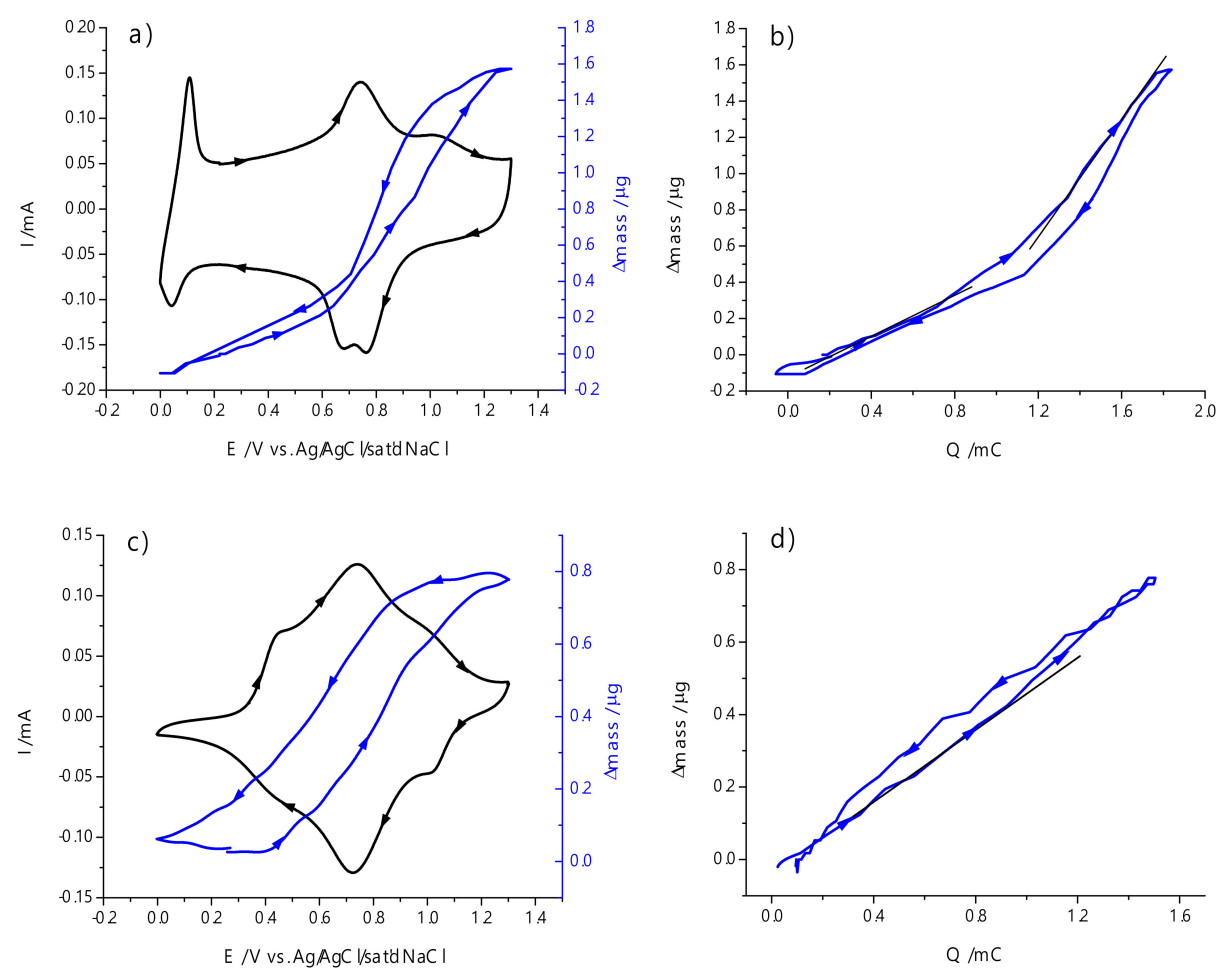
The change in the polymer film mass as a function of potential (blue line), cyclic voltammograms (black line) and corresponding Δ*m(q)* curves for poly-Ni(3-MeOSalen) (**a**,**b**) and poly-H_2_(3-MeOSalen) (**c**,**d**).

To maintain electroneutrality during electrochemical doping of the polymers, this process is accompanied by injection of charge compensating ions and solvent molecules. Sometimes, e.g., in self-doped polymers, charge-compensation is accomplished by expulsion of mobile ions trapped in the undoped polymer structure. We used EQCM technique to find molar mass of charge-transferring species participating in the polymer oxidation/reduction process and calculate the number of electrons exchanged during redox processes (Table 2 and Figure 5).

To calculate the number of electrons exchanged during redox processes (n), the cathodic part of the voltammetric curve was integrated to obtain the amount of charge passed in the electrochemical reduction of the polymer films (∆Q). Based on both Faraday law and the Sauerbrey equation [54], the relationship between the quantity of electricity and the number of electrons can be written as:

(1) $$n=\frac{\Delta Q\cdot M}{F\cdot \Delta m}$$

where ∆Q is the amount of charge passed in the electrochemical reduction of the polymer film (C), M is the molar mass of a polymer fragment (g mol^−1^), F is the Faraday constant (C mol^−1^), ∆m is a mass of the dried polymer film deposited on the electrode and measured by EQCM method (g).

Calculated values of the number of electrons per a monomer unit are presented in Table 2. Both the polymeric nickel complex poly-Ni(3-MeOSalen) and the polymeric ligand poly-H_2_(3-MeOSalen) comprise two redox active phenyl rings. Each of them can theoretically be oxidized by one electron. However, the number of electrons exchanged by poly-Ni(3-MeOSalen) film in the redox processes is less than two, and this value is slightly above one for the poly-H_2_(3-MeOSalen) film. Thus, not all units can be completely oxidized; some of them could be unavailable for oxidation or lose only one electron. Note that the presence of the metal ion in the monomer units of the film increases the number of electrons exchanged in redox processes of polymers.

The mass response of poly-Ni(3-MeOSalen) film is presented in Figure 5a,b. The mass of the polymer film increases by 1.6 µg during the anodic sweep and decreases by approximately the same amount in the reverse sweep. This indicates that the involved processes are reversible. For poly-Ni(3-MeOSalen) film, Δm(q) curve shows two linear regions. The potential ranges of certain type of counterion transfer coincide with the potential ranges of different types of oxidative processes in the polymeric film. In the potential range of cation radicals formation (0 - 0.77) V, the counterion molar mass is 45 g mol^−1^. This value can be attributed to the insertion of one BF_4_^-^ anion (87 g mol^−1^) per monomer unit to electrostatically compensate the formed positive charge and the expulsion of one molecule of acetonitrile (41 g mol^−1^) from the polymer film.

In the potential range of dication formation (0.77–1.30) V, the second linear region of Δm(q) curve is observed. The molar mass of the charge-compensating ion in the second redox process in the film is 147 g mol^−1^, which is equal to one BF_4_^-^ anion and 1.5 solvent molecules per one electron transfer. The presence of two slopes in Δm(q) curve confirms the existence of two different types of charge carriers: cation radicals and dications.

The voltammogram of the polymeric ligand poly-H_2_(3-MeOSalen) shows three oxidative processes but only one type of counterion transfer can be observed in the range of poly-ligand electroactivity (Figure 5c,d). Serious overlapping of voltammetric peaks indicates that several electrochemical processes in the film proceed in parallel and cannot be precisely resolved. Thus, the calculated molar mass of charge-transferring species shown in Table 2 can be considered as an averaged value. The mass of the polymer film increases by 0.8 µg during the anodic potential sweep and decreases by approximately the same amount during the reverse cathodic sweep, that is, the involved processes are reversible.

### 2.4. In Situ Electronic Spectra of Poly-Ni(3-MeOSalen) and Poly-H_2_(3-MeOSalen) Films

Polymeric complexes of Schiff bases are electrochromic materials. In situ spectroelectrochemical techniques can be used to obtain detailed information on the doping level, the nature of charged species, and the polymer structure in all oxidation states. In situ UV–VIS–NIR characteristic absorption spectrum of poly-Ni(3-MeOSalen) in 0.1 mol dm^−3^ Et_4_NBF_4_/AN electrolyte has been previously published and discussed in detail [24], so we have not focused on its interpretation and analysis. Briefly, consecutive generation of cation-radicals and dications in biphenoxyl fragments during electrochemical oxidation has been observed. Nickel metal centers act as non-electroactive but electron-coupling units. However, the in situ spectrum of the ligand poly-H_2_(3-MeOSalen) is novel and should be examined thoroughly.

**Figure 6:**
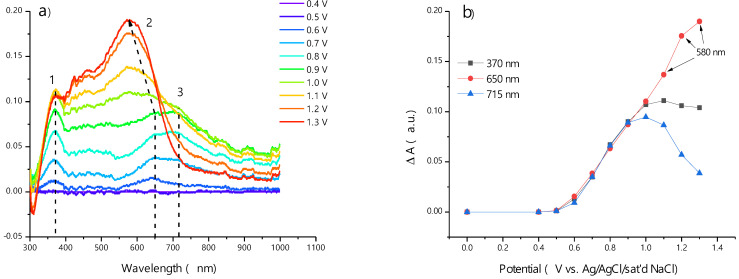
Evolution of in situ UV–VIS spectra during electrochemical oxidation of poly-H_2_(3-MeOSalen) in 0.1 mol dm^−3^ Et_4_NBF_4_/AN electrolyte, each UV–VIS spectrum was collected relative to that of the neutral (non-charged) polymer at the initial potential (**a**), and potential dependence of absorbance (**b**).

In situ absorption spectra were registered during potentiostatic polarization and presented in Figure 6a. At the beginning of poly-ligand oxidation (at 0.60 V) the growth of two bands at 370 nm (1), and 650 nm (2) is observed. These bands are typical for biphenoxyl cation radical formation [55,56,57]. The development of these bands during polymer electrooxidation indicates the formation of cation radicals in biphenyl fragments and an increase in their concentration in the potential range of 0.60–0.90 V. The growth of a broad NIR absorption with a pronounced peak at 715 nm (3) is observed in the potential range of 0.70–0.90 V. These spectral changes can be assigned to the formation of π-mers (dimer cation radicals) [58,59].

**Scheme 3:**
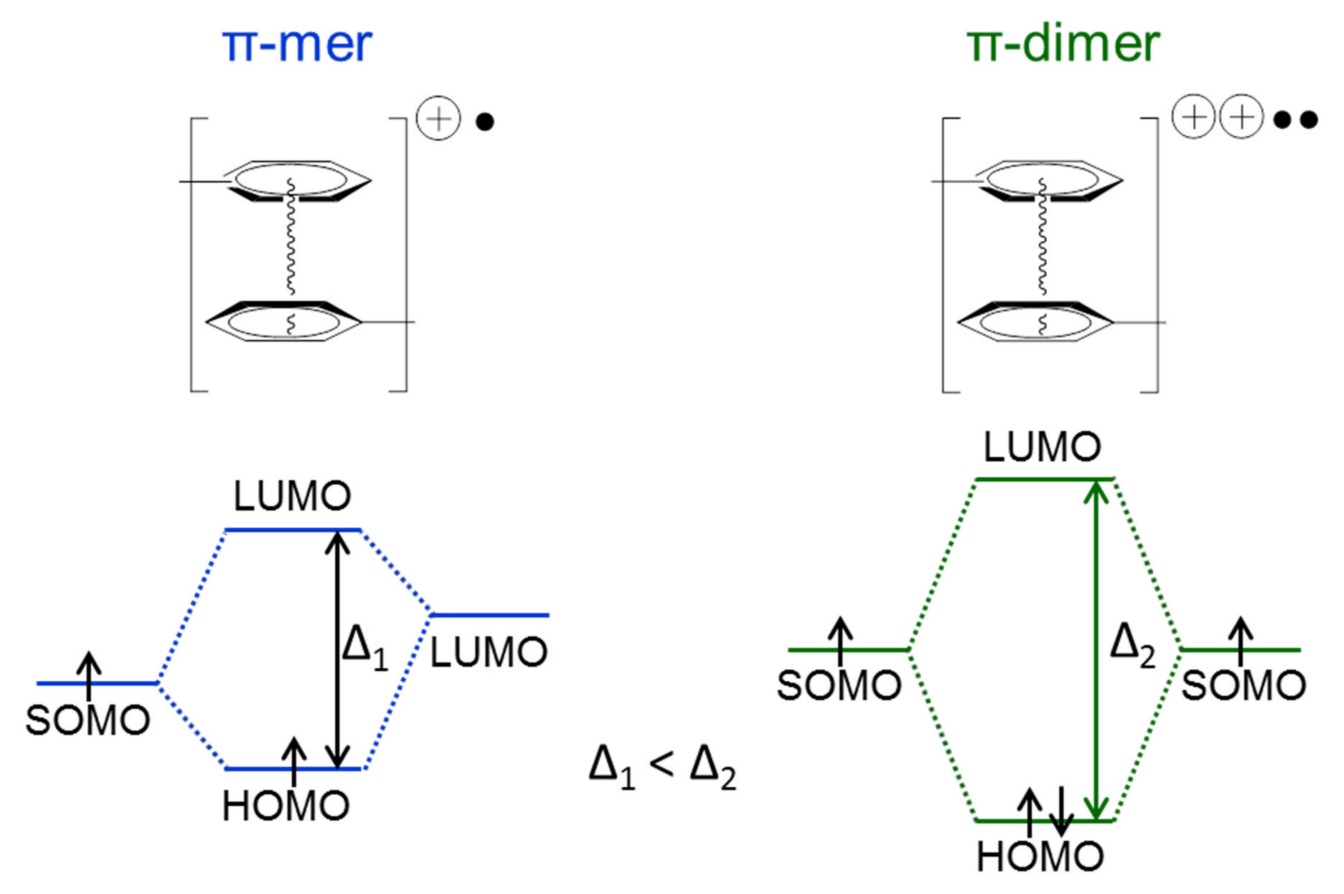
Schematic drawing of π-dimerization in a general case. Perturbation diagrams showing mixing of SOMO and LUMO to form new orbitals of π-mer (blue) and π-dimer (green). Black arrows indicate the spin of an electron in the corresponding orbital. The delta value characterizes the difference in energy between HOMO and LUMO.

The π-mers are formed by the frontier-orbital interactions of the SOMO–LUMO of poly-ligand fragments. The SOMO–LUMO interaction between the cation radical and the neutral counterpart (biphenolate moiety) causes a split in the HOMO and LUMO of the π-mer, and the band corresponding to the electronic HOMO–LUMO transition of the π-mer is observed [58] The mechanism of formation of such structures in a general case for the phenyl moieties is presented in the Scheme 3. The formation of π–π bonded radicals can be confirmed by absorption in the wavelength range of 400–550 nm between main absorption bands of biphenyl radicals. Such changes are typical for π-mers [58,60] and may be assigned to the vibrational structure of π–π bond between face-to-face arranged (bi)phenolate rings. A linear increase in the intensity of the abovementioned bands in the potential range of 0.50–0.90 V indicates an increase in the π-mers concentration in the polymer film (Figure 6b).

A spectrum of π-mer structures usually has an additional absorption band in the NIR region [61,62], but the poly-ligand spectrum shows the absorption band of a monomer cation radical (not assembled into a π-mer) in the same wavelength range, so the broad band (3) consists of two different overlapped bands. Consequently, at the low levels of oxidation (up to 0.70 V), simultaneous formation of monomer cation radicals and π-mers takes place, and the concentrations of these species increase with increasing electrode potential.

At higher levels of oxidation (above 0.90 V), the behavior of absorption bands changes. The intensities of the bands at 370 nm (1) and 650 nm (2) continue to rise up to 1.10 V while broad NIR absorption and the absorption at 715 nm (3) remain almost constant. Further doping up to 1.10 V leads to the appearance of the isosbestic point at 670 nm, significant reduction of NIR adsorption, and a blue shift and an increase in the intensity of the band (2). The blue shift of the band (2) with increasing potential is characteristic for π-dimer formation [63]. A π-dimer can be described as an assembly of two face-to-face cation radicals. Its SOMO–SOMO interactions cause the appearance of HOMO and LUMO electronic transitions in the π-dimer (Scheme 3) [64,65]. In the case of the poly-H_2_(3-MeOSalen) polymer, π-dimerization may happen between fragments of adjacent polymer chains located one above the other. A π-dimer is a stabilized species. The main stabilizing factor is the strength of SOMO–SOMO interactions, which depends on the charge delocalization in the polymer chain [64]. Less charge delocalization causes higher stabilization of π-dimers. The blue shift of the band (2) with increasing potential reveals that highly stable π-dimers are generated during oxidation. Thus, the conversion of π-mers to π-dimers occurs at high doping levels.

It should be noted that the polymer film poly-H_2_(3-MeOSalen) electrochemically reduced at the potential of 0 V has trapped charge. In the poly-ligand film spectrum equilibrated at 0 V the absorption band centered near 650 nm is observed. It can be related to the disproportionation of the π-mers into neutral and cationic (cation radical) species during polymerization [65]. In the absence of C-C bonding and interactions with other phenoxyl moieties, these cation radicals represent isolated charged units that are not in conjugation with the polymer backbone. Such “trapped” charge is stabilized by solvent molecules and interactions with counteranions.

### 2.5. The Morphology of the Films

**Figure 7:**
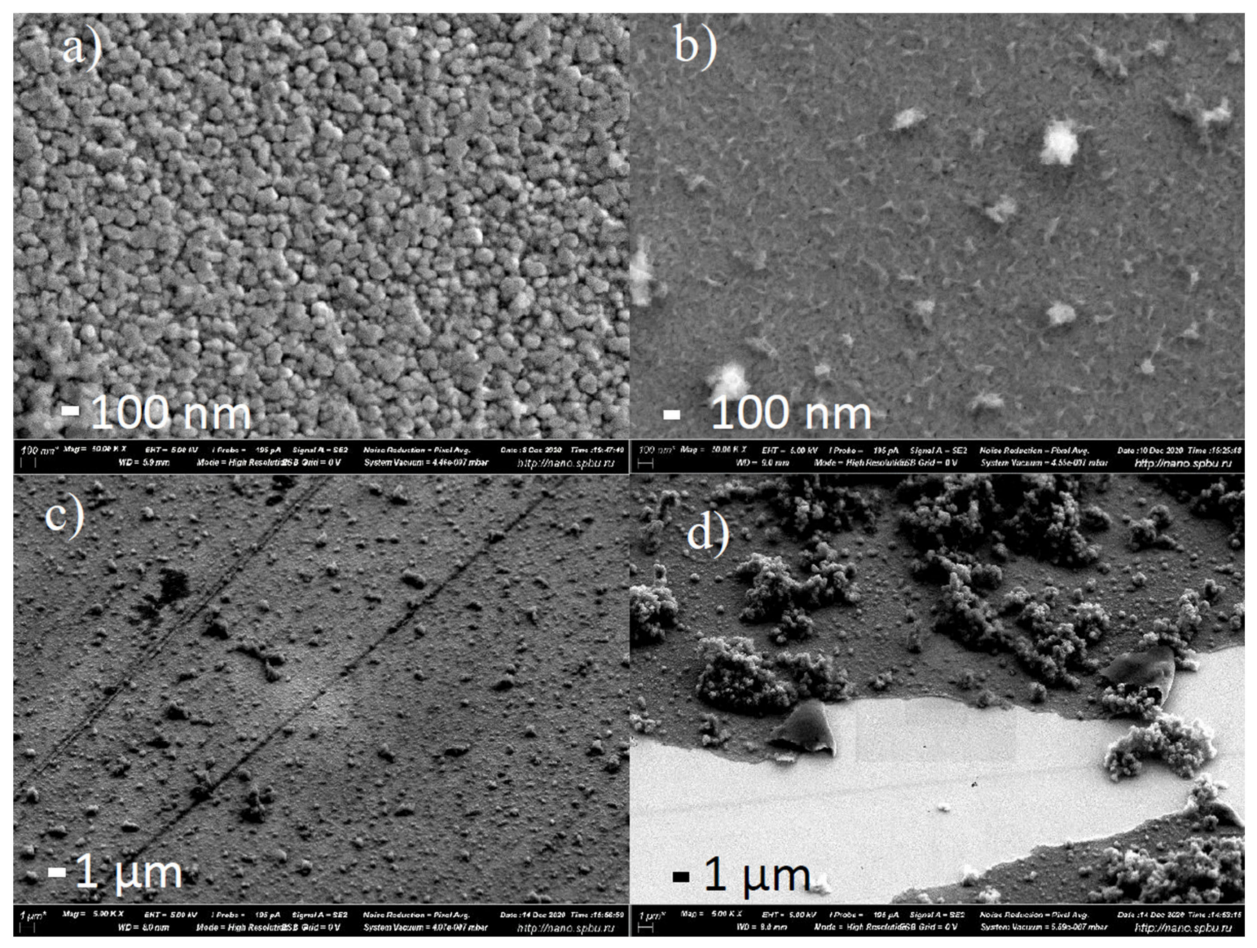
Scanning electron micrographs of polymer films poly-H_2_(3-MeOSalen) (**a**,**c**) and poly-Ni(3-MeOSalen) (**b**,**d**) deposited electrochemically on a platinum electrode by cycling from 0.0 to 1.0 V during 3 cycles (**a**,**b**) and 20 cycles (**c**,**d**).

The surface morphology of poly-H_2_(3-MeOSalen) and poly-Ni(3-MeOSalen) films of different thicknesses on Pt-electrodes is depicted in Figure 7. Thin polymer films were obtained by dynamic polymerization between 0.0 and 1.0 V during 3 cycles and their SEM images are presented in Figure 7a,b for the polymer films poly-H_2_(3-MeOSalen) and poly-Ni(3-MeOSalen), correspondingly.

As can be seen in Figure 7a, a thin polymer film poly-H_2_(3-MeOSalen) is closely packed in pea grains with small gaps between the particles, which allows electrolyte ions to penetrate into the entire polymer. Some of these grains are in contact with each other, but the others are separated by the electrolyte layer. This confirms the hypothesis that the film is primarily composed of dimeric structures rather than long polymer chain segments. Hence, we can expect the conductivity of the film as a whole to be due to the conductivity of individual grains that are not electrically connected to each other. The conductivity of such film is expected to be lower than that of the film exhibiting charge transport along polymer chains.

The introduction of nickel ions into the film structure leads to drastic changes in the morphology. The morphology of a thin polymer film poly-Ni(3-MeOSalen) is presented in Figure 7b. It shows a compact continuous structure and relatively smooth surface with small size globules sitting on top of a continuous layer. The film is permeated with a network of numerous pores, which allows electrolyte ions to move loosely in the film volume. The continuous film does not have isolated domains, which means that different areas of the polymer are electrically connected. The polymeric complex poly-Ni(3-MeOSalen) is capable of electron transferring both along polymer chains and between polymer chains and expected to possess sufficiently higher conductivity than the corresponding ligand.

The morphologies of thick films are significantly different. The 600 nm thick polymer film poly-H_2_(3-MeOSalen) is continuous, relatively dense, and does not show any noticeable pores (Figure 7c). The surface appears to be homogeneous. It has rather compact morphology and a low porosity. Moreover, the poly-H_2_(3-MeOSalen) film exhibits 450–500 nm sized spherical particles that are uniformly distributed on the dense film surface, with few areas showing agglomeration (Figure 7c).

Figure 7d shows the electron micrograph of a relatively thick polymer film poly-Ni(3-MeOSalen). The part of the polymer near the base has a tightly folded laminar layer of 100–150 nm in thickness. The corrugated film layer consists of randomly packed polymer aggregates enclosing pores. It grows along the base and can be up to 1–3 µm thick. Additionally, polymer films in whole have a porous structure that facilitates good contact of the electrolyte with the polymer. This two-phase structure implies that the polymerization process initially takes place on the electrode surface due to the presence of oligomers in solution that form a smooth compact layer. As the polymerization is continued, the polymer aggregates are formed due to the association of oligomers in solution and their rapid deposition on the growing film [66].

### 2.6. The Structure and Mechanism of Charge Transfer in the Polymer Films Poly-Ni(3-MeOSalen)

**Scheme 4:**
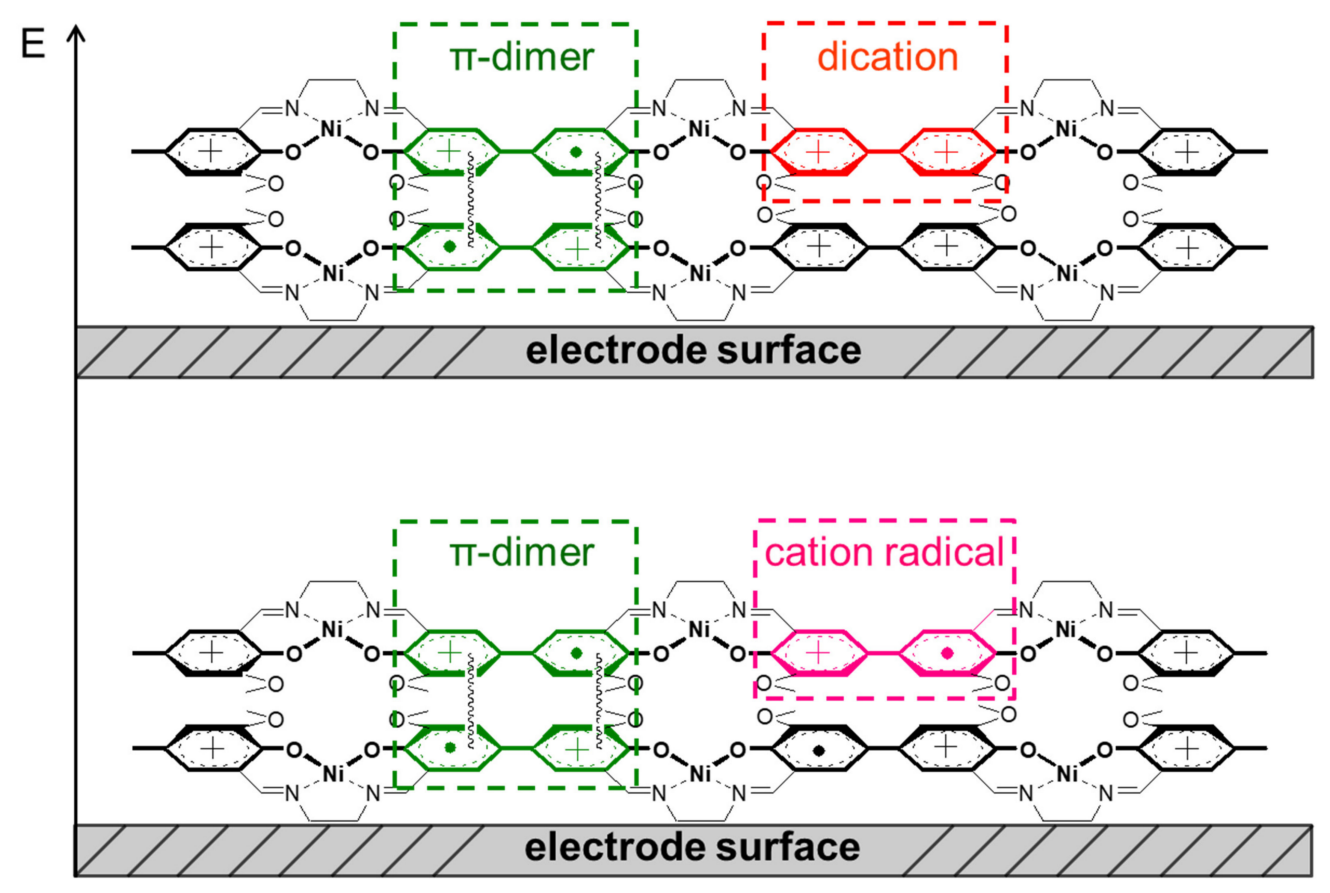
Schematic representation of polymer film poly-Ni(3-MeOSalen) at different doping levels. Depending on the doping level such charge carriers as cation radicals (pink), dications (red) and π-dimers (green) are formed.

At the beginning of poly-Ni(3-MeOSalen) film oxidation, the one electron transfer per monomer unit takes place and cation radicals are formed (Scheme 4). Cation radicals are localized on biphenyl fragments. The formation of cation radicals and an increase in their concentration with increasing electrode potential are confirmed by the appearance of corresponding bands in UV–VIS spectra and the growth of voltammetric currents. When the π-electronic systems of biphenyl rings are packed face-to-face, the electronic interactions between polymer chains occur and π-dimers are formed. These interactions are weak and strongly depend upon the degree of charge delocalization and the presence of bulky substituting groups [35]. The polymers with more localized cation radicals and without steric limitations are most suitable to produce π-dimers. 

Further oxidation of the polymer film leads to the second electron transfer per monomer unit and dications formation. This process is accompanied by the formation of a new band in the UV–VIS spectra and a decrease in the absorption intensity of the bands assigned to cation radicals. The maximum theoretical number of electrons exchanged during redox process equals two for poly-Ni(3-MeOSalen) (one electron per phenyl ring). However, the number of electrons estimated by EQCM method is lower and equals to 1.4 electrons per monomer unit.

**Scheme 5:**
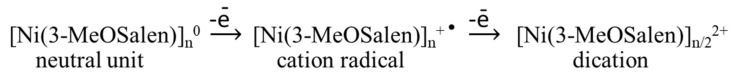
Schematic representation of oxidation of poly-Ni(3-MeOSalen).

This phenomenon is related to the presence of π-dimers formed at the stage of the first one electron transfer in the polymer film. π-Dimers are structures stabilized via π···π stacking between benzene rings. These structures can dissociate and undergo further oxidation forming two dications. Alternatively, they do not dissociate and are not further oxidized at these potentials. Based on the EQCM-estimated doping level, we can assume that only half of cation radicals transform into dications (Scheme 5).

The monomer complex Ni(3-MeOSalen) has a square planar molecular geometry in the coordination sphere that allows it to form dense ordered films during polymerization. The central nickel ion plays a role of the bridge that mediates the coupling between two redox-active phenolates. On the one hand, this helps to stabilize the forming radical-type sites by the metal-to-ligand system. On the other hand, this provides electron transfer between the neighboring monomeric units in the polymer film. As a result, the main way of electron transfer in the polymer film poly-Ni(3-MeOSalen) is along the polymer chain, while redox processes are ligand-based.

### 2.7. The Structure and Mechanism of Charge Transfer in the Polymer Films Poly-H_2_(3-MeOSalen)

**Scheme 6:**
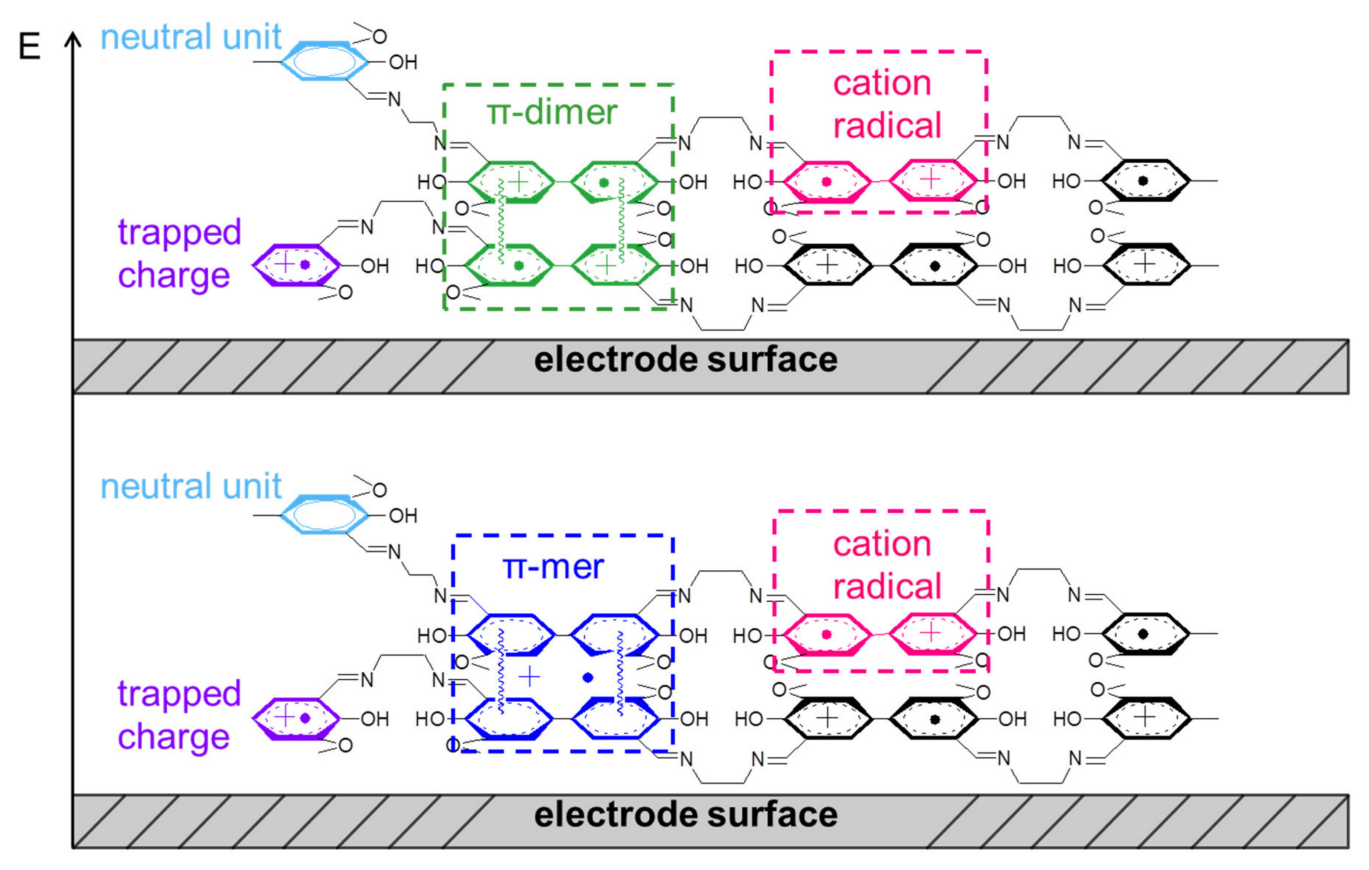
Schematic representation of the polymer film poly-H_2_(3-MeOSalen) at different doping levels. At a low doping level the main charge carriers in the film are cation radicals (pink) and π-mer structures (blue). With an increase in the doping level, the conversion of π-mers (blue) to π-dimers (green) occurs. Defects in the polymer structure such as “trapped” charge (purple) and neutral units (baby blue) are not involved in redox processes. The deviation of monomeric units from planarity was ignored for clarity.

The structure and, consequently, the mechanism of charge transfer in the polymeric ligand poly-H_2_(3-MeOSalen) are markedly different from the corresponding polymeric nickel complex (Scheme 6). First of all, this is related to the planarity of monomeric compounds: deviation from planarity of N_2_O_2_ sphere is 65° in H_2_(3-MeOSalen) [67]. As a result, these monomers form less ordered films. In poly-H_2_(3-MeOSalen) films, the monomer units are jointed together both by C-C bonds between aromatic rings of neighboring molecules and by intermolecular π–π interactions of face-to-face packed phenolate moieties. In addition, there are defects in the film structure: (a) “trapped charge”—the structures representing isolated charged units that can formed during polymerization process as a result of π-mers disproportionation but are not conjugated with the polymer backbone, and (b) neutral units—phenolate moieties that do not participate in conjugation.

During the first redox process in the poly-H_2_(3-MeOSalen) film, simultaneous formation of biphenyl cation radicals (similar to those generated in non-interacting chain segments of poly-Ni(3-MeOSalen) upon oxidation) and π-mers (cation radicals delocalized between two stacked phenolate moieties) occurs. These processes are clearly manifested by an increase in the absorption intensity of corresponding bands in the UV–VIS spectra. The second one electron transfer step leads to the formation of π-dimers (dication diradicals delocalized between two stacked phenolate moieties), this process shows itself in the UV–VIS spectra as a shift in the corresponding band to lower wavelengths with increasing absorption intensity. π-Dimers can consist of two biphenyl or two mono-phenyl stacked structures and exchange one or two electrons per phenyl ring, respectively (Scheme 6). So, one part of the film can exchange two electrons and the other part can exchange only one electron. Experimentally estimated doping level is slightly above one. However, we note that the mass of the electroactive polymer on the electrode surface is likely overestimated due to the existence of defects in the polymer structure such as “trapped” charge and neutral units. It is certain that there are a fairly large number of mono-phenyl stacked structures in the polymer film.

**Scheme 7:**
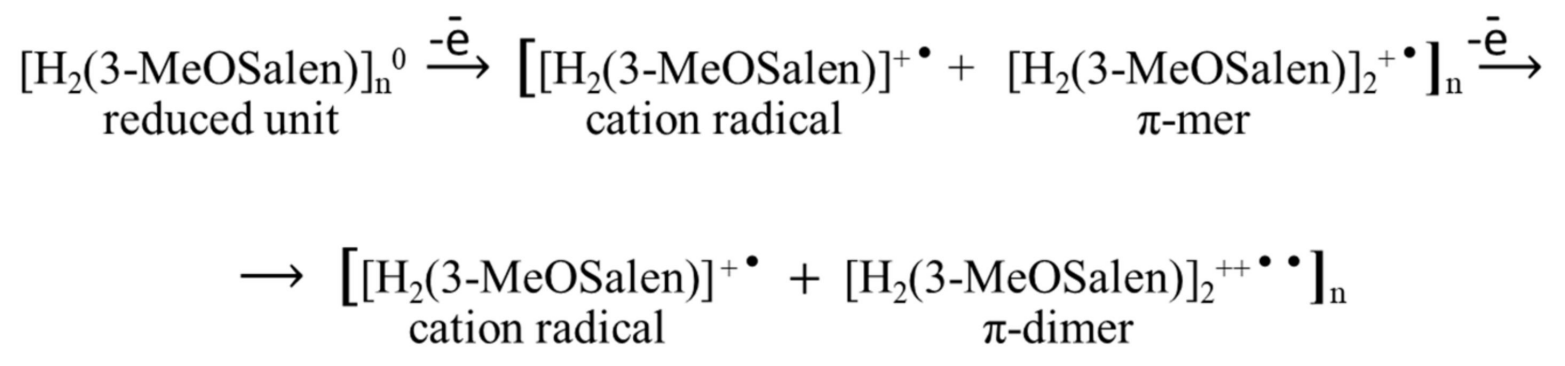
Schematic representation of oxidation of the poly-H_2_(3-MeOSalen).

So, the main pathway of electron transfer in the polymer film poly-H_2_(3-MeOSalen) is between π-stacked structures and C-C bonded phenyl rings. Polymer chains consist of separated biphenyl fragments with no conjugation along the chains. Bulk electron transfer occurs only by interchain interactions in π-dimers (Scheme 7). A highly defective and disordered polymer structure prevents the formation of thick polymer films.

## 3. Materials and Methods

### 3.1. Chemicals

3-methoxysalicylaldehyde (Sigma-Aldrich, Saint Louis, MO, USA, 99%), ethylenediamine (Sigma-Aldrich, 99%) and nickel(II) acetate tetrahydrate Ni(AcO_2_) 4H_2_O (analytical grade) were used as received. The monomeric complex N,N’-ethylene-bis(3-methoxysalicylideneiminato) nickel(II)(Ni(3-MeOSalen)) and the corresponding ligand N,N’-bis(3-methoxysalicylidene) ethylenediamine (H_2_(3-MeOSalen)) were synthesized as described in work [68]. Acetonitrile (anhydrous, J.T. Baker, less than 30 ppm water) was used without further purification. Tetraethylammonium tetrafluoroborate (Et_4_NBF_4_, Sigma-Aldrich, Saint Louis, MO, USA, 99%) was dried at 65 °C for 72 h before use. All solutions were prepared in a dry glove box under an inert atmosphere of argon before the measurements.

### 3.2. Electrochemistry and Electrochemical Quartz Crystal Microbalance (EQCM)

Potentiostat (BioLogic Science Instruments, Seyssinet-Pariset, France) and 5 MHz QCM100 Quartz Crystal Microbalance (Stanford Research Systems, Sunnyvale, CA, USA) connected to a Metex MXC 1600 frequency counter (Metex Co, Seoul, Korea) were used for electrochemical measurements and the determination of the deposited polymers mass. Electrochemical measurements were performed in a three-electrode cell with a platinum-coated quartz piezoelectric crystal (electrode area 1.37 cm^2^) as the working electrode, a glassy carbon plate (electrode area 12.5 cm^2^) as the counter electrode and a non-aqueous Ag/Ag^+^ reference electrode (MW-1085, BASi) filled with a 0.005 mol dm^−3^ AgNO_3_ solution in 0.1 mol dm^−3^ Et_4_NBF_4_/acetonitrile. The potential of this reference electrode was −0.30 V vs. Ag/AgCl/sat’d NaCl. All potentials in this work were referred to Ag/AgCl/sat’d NaCl reference electrode.

Polymer deposition was performed in 0.001 mol dm^−3^ monomer/acetonitrile solution with 0.1 mol dm^−3^ Et_4_NBF_4_ as the supporting electrolyte by cycling the potential of the working electrode between 0 and 1.2 V. The scan rates for deposition and the number of polymerization cycles were adjusted for each monomer to produce approximately 4 µg of the dry polymer on the electrode surface. After that, the electrode with the deposited polymeric film was rinsed with acetonitrile and dried in argon for 30 min. The mass of the dried polymer was estimated using the Sauerbrey equation [54]. The electrode was then transferred to the supporting electrolyte solution (0.1 mol dm^−3^ Et_4_NBF_4_ in acetonitrile) and electrochemical measurements were performed in the potential range of 0–1.2 V at 0.05 V s^−1^.

To confirm that the polymeric films were fully oxidized and reduced, the cyclic voltammetry measurements at different scan rates were performed. The linear dependence of voltammetric peak currents versus scan rates indicated that films operated under the conditions of thin layer voltammetry.

### 3.3. In Situ UV–VIS Spectroelectrochemistry

In situ UV–VIS absorption spectra of polymers were registered using an SF-2000 spectrophotometer (Spectr, Saint Petersburg, Russia) in a dry glove box under an inert atmosphere of argon.

The spectroelectrochemical measurements of polymer films were carried out in a quartz cuvette, with indium tin oxide-coated glass (ITO) as the working electrode (electrode area 1 cm^2^), a platinum wire as the counter electrode and a silver chloride-coated silver wire as the pseudo-reference electrode. The potential of the pseudo-reference electrode was very close to that of Ag/AgCl/sat’d NaCl electrode, so all potentials were referred to Ag/AgCl/sat’d NaCl electrode.

The polymer films were deposited on the ITO electrode (Sigma Aldrich, electrode area 1.0 cm^2^) from the acetonitrile solution containing the monomer (concentration 0.001 mol dm^−3^) and the supporting electrolyte (0.1 mol dm^−3^ Et_4_NBF_4_). The electropolymerization was carried out by potential cycling in the range of 0–1.2 V at 0.05 V s^−1^.

UV–VIS spectra of polymer films were registered in situ at various applied potentials corresponding to the films in different oxidation states. Spectra were recorded in the range of 300–1000 nm at fixed potentials during stepwise oxidation from 0 to 1.30 V. All spectra were recorded after reaching the equilibrium state.

### 3.4. FTIR Spectroscopy

IR spectra of monomers Ni(3-MeOSalen) and H_2_(3-MeOSalen) were recorded using the standard KBr pellet technique on the Shimadzu IRPrestige-21 spectrometer (Shimadzu Corporation, Tokyo, Japan). IR spectra of corresponding polymeric films were recorded using the diffuse reflection technique. The electrodes modified with polymer films were obtained using the same procedure as was used to prepare electrodes for UV–VIS spectroscopic measurements. Modified electrodes were rinsed with acetonitrile and dried under an inert atmosphere of argon before spectroscopic measurements.

### 3.5. Scanning Electron Microscopy

The morphologies of poly-Ni(3-MeOSalen) and poly-H_2_(3-MeOSalen) films were evaluated by scanning electron microscopy (SEM). The scanning electron microscope (Zeiss Merlin, Jena, Germany) with a 5 kV accelerating voltage was used. The investigated films were formed on the platinum surface by cycling the potential of the platinum electrode immersed in an acetonitrile solution. The solution composition and deposition parameters (potential range, sweep rate) were the same as for EQCM measurements. The number of polymerization cycles was varied to obtain polymeric films of different thickness (3 or 20 cycles). The thicknesses of the polymer films were ca. 150 nm and a few microns for the thin (3 cycles) and the thick (20 cycles) films, respectively, as determined from the cross-section views of the specimen. After deposition, the polymeric films were washed thoroughly with acetonitrile and dried under argon atmosphere.

## 4. Conclusions

Polymer films of the Salen-type ligand poly-H_2_(3-MeOSalen) and the corresponding metallopolymer poly-Ni(3-MeOSalen) were investigated by electrochemical and spectral methods. Based on the comparative analysis, the role of the central metal atom in the charge transport and its impact on the structure of the forming polymer films were established.

Based on FTIR, voltammetry and EQCM studies, the relationships between the monomer structure and the structures of the forming polymer films have been shown. In the case of the polymeric ligand poly-H_2_(3-MeOSalen), the corresponding monomer molecules have noticeable deviation from planarity and form more disordered polymer films. The polymer film consists of C-C bonded phenoxyl moieties and π-stacked species and also has defects such as “trapped” charge and neutral units. At the low doping level, there are biphenyl cation radicals and π-mers in the polymer film; further oxidation results in the appearance of π-dimers. The main pathway of electron transfer in the polymer film poly-H_2_(3-MeOSalen) is between π-stacked structures and C-C bonded phenyl rings.

Owing to the presence of the metal ion in the monomer structure, the complex Ni(3-MeOSalen) has square planar geometry that allows to form highly ordered, dense films with long polymer chains. These chains interact with each other forming π-stacked species. Such interactions are weak but promote additional stabilization of polymeric films. Oxidation of the films poly-Ni(3-MeOSalen) is accompanied by cation radicals formation, further oxidation leads to dications. The main pathway of electron transfer in the polymer film poly-Ni(3-MeOSalen) is along polymer chains.

The reversible redox process in poly-H_2_(3-MeOSalen) may be of interest for electrochemical energy storage devices. As an organic material readily available from simple molecular buildings blocks by electrooxidative polymerization and possessing an exclusively intermolecular charge transfer mechanism, poly-H_2_(3-MeOSalen) also seems to be a promising material for electronics and photonics.

## Figures and Tables

**Table 1:** Infrared spectra frequencies (cm^−1^) for the investigated monomeric and polymeric compounds.

Compound	ν(C=N), cm^−1^	ν(C-O), cm^−1^	ν(C-N), cm^−1^	ν(O-H), cm^−1^
H_2_(3-MeOSalen)	1633(s)	1250(s)	1409(w)	2600
Ni(3-MeOSalen)	1622(s)	1315(s)	1411(w)	-
poly-H_2_(3-MeOSalen)	1628(s)	1250(s)	1409(w)	2600
poly-Ni(3-MeOSalen)	1617(s)	1315(s)	1405(w)	-

**Table 2:** Molar mass of charge-transferring species participating in the polymer oxidation/reduction process and the number of electrons per monomer unit exchanged during redox processes in the polymer films.

	Poly-Ni(3-MeOSalen)		Poly-H_2_(3-MeOSalen)
Potential range, V	0 ÷ 0.77	0.77 ÷ 1.30	0 ÷ 1.30
Molar mass of charge-transferring species, g mol^−1^	45 ± 5	147 ± 12	46 ± 4
Number of electrons per a monomer unit	1.4 ± 0.1		1.1 ± 0.1

## Data Availability

The data presented in this study are available on request from the corresponding author.
